# Anxiety and Depression in Cervical Cancer Patients in Relation to Nutritional Status and Oxidative Stress

**DOI:** 10.3390/medsci14060657

**Published:** 2026-10-09

**Authors:** Ioana-Sadiye Scripcariu, Daniela Roxana Matasariu, Demetra Gabriela Socolov, Gabriel-Ioan Anton, Raluca Anca Balan, Ramona Gabriela Ursu, Viorel Dragos Radu, Andreea-Luciana Avasiloaiei, Cătălina Ionescu, Mălina Visternicu, Alin Ciobica, Bianca Buzilă (Platon), Ciprian Ilea, Mihaela Tomaziu-Todosia, Ecaterina Tomaziu-Todosia Anton

**Affiliations:** 1 Grigore T. Popa University of Medicine and Pharmacy, University Street, No. 16, 700115 Iasi, Romania; ioana.scripcariu@umfiasi.ro (I.-S.S.); daniela.matasariu@umfiasi.ro (D.R.M.); demetra.socolov@umfiasi.ro (D.G.S.); anton.gabriel-ioan@d.umfiasi.ro (G.-I.A.); raluca.balan@umfiasi.ro (R.A.B.); ramona.ursu@umfiasi.ro (R.G.U.); viorel.radu@umfiasi.ro (V.D.R.); andreea.avasiloaiei@umfiasi.ro (A.-L.A.); bianca_buzila@email.umfiasi.ro (B.B.); ilea.ciprian@umfiasi.ro (C.I.); tomaziu-todosia.ecaterina@d.umfiasi.ro (E.T.-T.A.); 2 Department of Obstetrics and Gynecology, Clinical Hospital of Obstetrics and Gynecology Cuza Voda, Cuza Voda Street, No. 34, 700038 Iasi, Romania; 3 “C.I. Parhon” University Hospital, 700503 Iasi, Romania; 4 Doctoral School of Biology, Faculty of Biology, “Alexandru Ioan Cuza” University of Iasi, 700505 Iasi, Romania; catalinaionescu81@yahoo.com (C.I.); malina.visternicu@yahoo.ro (M.V.); 5 “Ioan Haulica” Institute, Apollonia University, 700511 Iasi, Romania; alin.ciobica@uaic.ro; 6 Department of Biology, Faculty of Biology, “Alexandru Ioan Cuza” University of Iasi, 700505 Iasi, Romania; 7 “Olga Necrasov” Center, Department of Biomedical Research, Romanian Academy, 700506 Iasi, Romania

**Keywords:** cervical cancer, anxiety, depression, nutritional status, oxidative stress, psychological distress, quality of life

## Abstract

Cervical cancer remains a major global health concern, causing substantial morbidity and mortality, particularly in low- and middle-income countries. Beyond its physical burden, patients frequently experience significant psychological distress, including anxiety and depression, which can adversely affect treatment adherence, daily functioning, and overall quality of life. Emerging evidence highlights the intricate relationships between psychological well-being, nutritional status, and oxidative stress in this population. Malnutrition and cancer cachexia may exacerbate fatigue, cognitive dysfunction, and emotional vulnerability, while oxidative stress contributes both to disease progression and neuropsychological disturbances. This narrative review synthesizes current research on anxiety and depression in cervical cancer patients, emphasizing how impaired nutritional status and elevated oxidative stress are associated with increased psychological burden. Understanding these interconnections is essential for developing comprehensive, multidisciplinary interventions that address both somatic and emotional health, optimize patient outcomes, and improve quality of life.

## 1. Introduction

Cervical cancer is among the most common cancers affecting women globally, causing considerable morbidity and mortality despite improvements in early detection and therapeutic approaches [1,2]. According to the latest GLOBOCAN estimates, approximately 604,000 new cases of cervical cancer and 280,000 related deaths occurred worldwide in 2024, with a disproportionate burden persisting in transitioning countries [3], the highest burden being observed in low- and middle-income countries [4].

Beyond its physical burden, cervical cancer also imposes significant psychological challenges. Anxiety and depression are commonly observed, often shaped by factors such as disease stage at diagnosis, treatment modalities, and concerns related to fertility and body image [5,6,7]. These mental health issues not only amplify the overall disease burden but can also interfere with treatment adherence, daily functioning, and overall quality of life, creating a cycle in which physical and psychological consequences reinforce one another. Recognizing this interplay is essential for delivering comprehensive care that addresses both the somatic and emotional needs of patients [8,9]. Beyond these general psychological manifestations, women with cervical cancer may experience specific psychosexual concerns, including impaired sexual function, dyspareunia, vaginal dryness or stenosis, altered body image and perceived femininity, and distress related to fertility loss or reduced reproductive potential. These concerns are particularly relevant because radical surgery and pelvic radiotherapy can directly affect reproductive and sexual function. Moreover, the etiological association of cervical cancer with persistent high-risk human papillomavirus (HPV) infection may contribute to stigma, concerns regarding intimate relationships, and additional emotional distress [10,11,12].

Emerging evidence suggests a complex interplay between nutritional status, oxidative stress, and psychological well-being in cervical cancer patients [13,14,15,16]. Malnutrition and cancer-related cachexia may worsen fatigue, cognitive function, and emotional resilience, while oxidative stress can contribute to both disease progression and neuropsychological disturbances [17,18,19]. Treatments such as surgery, chemotherapy, and radiotherapy, while effective against the tumor, frequently produce side effects that interfere with daily functioning [20,21].

Previous reviews have generally addressed psychological distress, nutritional factors, and oxidative stress in cervical cancer as separate or partially overlapping domains. The distinctive contribution of the present review is to integrate these dimensions within a unified perspective, with particular emphasis on their potential bidirectional interactions. Rather than considering anxiety and depression solely as consequences of cancer diagnosis and treatment, we examine how nutritional impairment, systemic and metabolic alterations, and oxidative imbalance may contribute to psychological vulnerability, while psychological distress may, in turn, adversely affect dietary intake, treatment adherence, and overall functional status. This integrative perspective may provide a more comprehensive understanding of the mechanisms contributing to psychological distress in patients with cervical cancer and identify clinically relevant targets for multidisciplinary management.

This narrative review aims to provide a comprehensive overview of anxiety and depression in cervical cancer patients, with particular focus on the role of nutritional status and oxidative stress. By synthesizing current evidence, we seek to highlight potential mechanisms, identify gaps in knowledge, and inform strategies for multidisciplinary interventions that address both physical and psychological needs.

## 2. Literature Search Methodology

A narrative literature search was conducted between March and August 2026 using PubMed, ScienceDirect, Scopus, and Web of Science. No predefined publication-date restriction was applied. The search strategy used combinations of keywords related to the three main domains of the review. Search terms included “cervical cancer”, “cervical carcinoma”, “cervical neoplasia”, and “human papillomavirus (HPV)”, combined with terms related to psychological outcomes (“anxiety”, “depression”, “psychological distress”, “mental health”), nutritional factors (“nutrition”, “nutritional status”, “malnutrition”, “cachexia”, “diet”, “dietary factors”, “micronutrients”, “vitamins”, “minerals”), and oxidative stress (“oxidative stress”, “reactive oxygen species”, “ROS”, “antioxidants”, and “oxidative stress biomarkers”).

Studies were considered eligible when they addressed at least one of the three main domains of the review, psychological distress, nutritional status, or oxidative stress, in relation to cervical cancer, cervical intraepithelial neoplasia, or HPV-related cervical disease. Priority was given to studies conducted specifically in cervical cancer populations. Because direct evidence linking nutritional status and oxidative stress with psychological outcomes in cervical cancer remains limited, evidence from broader oncological populations was included when relevant to the proposed biological or clinical relationships; such evidence is explicitly identified in the manuscript as indirect or extrapolated evidence. Original studies, systematic reviews, meta-analyses, and relevant narrative reviews published in English were considered. Studies without relevance to the predefined domains, publications for which sufficient information was unavailable, and duplicate records were excluded.

Given the narrative nature of the review, study selection was based on relevance to the predefined thematic domains rather than on a formal systematic-review protocol. The literature was critically synthesized with attention to study design, population characteristics, assessment methods, consistency of findings, and the distinction between direct cervical cancer-specific evidence and evidence extrapolated from other populations.

## 3. Psychological Distress in Cervical Cancer Patients

### 3.1. Prevalence of Anxiety and Depression

Mental disorders have been investigated in relation to cervical cancer risk; however, the observed associations appear to be complex and may be influenced by psychosocial factors, health-related behaviors, and differences in access to preventive care and screening [22,23]. For instance, dysregulation of immune function and hormonal imbalances associated with mood disorders may influence carcinogenesis, while women with mental disorders often experience lower rates of cervical cancer screening, which can increase disease risk [23,24]. Although mood disorders, such as depression, anxiety, schizophrenia, and bipolar disorder, are among the most prevalent mental conditions in women, their impact on cervical cancer development remains unclear [25,26]. Some cohort studies suggest that certain disorders, like schizophrenia or bipolar disorder, may be associated with higher cervical cancer incidence, whereas findings for depression and anxiety are inconsistent [27,28,29]. Symptoms often appear soon after diagnosis and may persist throughout treatment and into survivorship. Moreover, these relationships appear to vary across populations, ethnicities, and geographic regions, underscoring the complexity of the interplay between mental health and cancer risk.

Anxiety can manifest as persistent worry about disease progression, treatment side effects, and concerns regarding fertility or sexual function, while depression is often characterized by low mood, fatigue, and decreased motivation [30,31].

As presented in Table 1, multiple studies have investigated the prevalence and determinants of anxiety in patients with cervical cancer, consistently highlighting its significant burden.

Zhao et al. (2020) [32] conducted an observational case–control study including 158 cervical cancer patients and 150 healthy controls, using the Hospital Anxiety and Depression Scale (HADS). Their findings demonstrated that anxiety levels were significantly higher among patients compared to controls and were also associated with poorer survival outcomes, suggesting a potential prognostic role of psychological distress. Similarly, Lu et al. (2019) [33], in a large cohort study involving 4245 patients, reported that psychological distress, including anxiety, was significantly associated with increased cancer-specific mortality. This large-scale analysis reinforces the importance of psychological factors as predictors of clinical outcomes. Li et al. (2023) [34] performed a cross-sectional study on 294 patients and found a high prevalence of anxiety and depression. Their results emphasized the role of psychosocial determinants, showing that factors such as social support and sense of coherence significantly influenced anxiety levels.

Another cross-sectional study focusing on quality of life parameters reported that approximately 60% of patients experienced anxiety, which was strongly associated with symptoms such as fatigue, insomnia, and impaired emotional functioning. These findings highlight the close relationship between psychological distress and overall quality of life in cervical cancer patients [35]. In addition, a study utilizing the Zung Self-Rating Anxiety Scale on 59 patients demonstrated that anxiety severity increased with advancing disease stage, indicating that disease progression may exacerbate psychological burden [37]. More recent evidence from a cross-sectional study (2024) showed that anxiety affected approximately 39.5% of patients undergoing radiotherapy and was significantly associated with illness uncertainty, further supporting the role of cognitive and emotional factors in cancer-related anxiety [36]. Finally, an observational study examining resilience and social support found that higher levels of these factors were associated with lower anxiety levels, suggesting that psychological and social resources may mitigate distress in this patient population [38].

Depression is one of the most common mood disorders observed in women with cervical cancer and can emerge at any point, from diagnosis through treatment and survivorship. It is typically characterized by persistent low mood, fatigue, loss of interest, and diminished motivation, which can significantly impair daily functioning and quality of life [39,40,41]. In the context of cervical cancer, depressive symptoms may be exacerbated by concerns about disease progression, treatment side effects, fertility, and sexual health. The presence of depression can also interfere with adherence to therapy, reduce engagement with follow-up care, and amplify physical symptoms, creating a cycle in which emotional distress and disease burden reinforce one another [42,43,44].

Taken together, these studies consistently indicate a substantial burden of depressive symptoms among patients with cervical cancer, although the reported prevalence and associated factors vary across populations. These differences may partly reflect methodological heterogeneity, including differences in sample size, disease stage, treatment phase, and psychological assessment instruments. Moreover, much of the available evidence is cross-sectional, allowing the identification of associations between depression and clinical or psychosocial factors but limiting conclusions regarding causality. In contrast, case–control and meta-analytic evidence strengthens the conclusion that depressive symptoms represent a clinically relevant problem in this population. Overall, the evidence appears more consistent regarding the presence and clinical significance of depression than regarding its precise prevalence or individual causal determinants.

As presented in Table 2, several studies have examined the prevalence of depression in patients with cervical cancer, consistently showing a high burden of depressive symptoms.

Zhao et al. (2020) [32], in an observational case–control study with 158 patients and 150 healthy controls, found that depression levels were significantly higher in cervical cancer patients compared to controls, and were also associated with poorer overall survival. Similarly, Golubovic et al. (2022) [37], using the Zung Self-Rating Depression Scale on 59 patients, reported that depressive symptoms increased with advancing disease stage, highlighting the impact of disease severity on psychological well-being.

In a cross-sectional study of 224 patients, Chinese researchers (2013) [6] reported a prevalence of depression of approximately 52.2%, with a high co-occurrence of anxiety, and found that psychosocial resources, including hope and optimism, were associated with lower depression scores. A meta-analysis by Zhang et al. (2026) [40], which included 5681 patients across 26 studies, reported a pooled prevalence of depressive symptoms around 46% during the peri-chemoradiotherapy period, emphasizing that depression is a common experience throughout treatment. Li et al. (2023) [34], in a cross-sectional study with 294 patients, demonstrated that higher perceived social support and a stronger sense of coherence were associated with lower depressive symptom severity.

### 3.2. Risk Factors

The development of anxiety and depression in cervical cancer patients is influenced by a multifaceted interplay of disease-related, demographic, psychosocial, and treatment-specific factors [37]. Among disease-specific variables, advanced tumor stage, a recent diagnosis, and the aggressiveness or complexity of treatment regimens, including surgery, radiotherapy, and chemotherapy, are consistently associated with higher psychological distress [46,47]. These factors not only reflect the perceived threat to survival but also amplify physical symptoms such as pain, fatigue, and functional limitations, which in turn contribute to emotional burden. Importantly, treatment-related psychological distress in cervical cancer may also be linked to changes that directly affect reproductive and sexual health. Radical surgery and pelvic radiotherapy may impair fertility and sexual function, while treatment-related vaginal changes, dyspareunia, and altered body image may further contribute to anxiety, depressive symptoms, and difficulties in intimate relationships.

Sociodemographic characteristics further modulate vulnerability to psychological distress. Younger patients may experience heightened anxiety due to concerns about fertility, sexual health, and long-term life plans, while individuals with limited social support or lower educational and socioeconomic status often face greater challenges in coping with the demands of disease and treatment [48,49,50]. Cultural norms and societal expectations regarding femininity, body image, and motherhood may also intensify emotional strain, particularly in patients undergoing radical procedures or facing visible physical changes [51,52]. These factors may be particularly relevant in younger women, for whom the consequences of treatment can interfere with reproductive plans, sexuality, intimate relationships, and family roles. Socioeconomic disadvantage may further increase psychological vulnerability by limiting access to supportive care and increasing the practical and financial burden associated with prolonged cancer treatment.

Additionally, pre-existing mental health conditions, personal coping styles, and resilience levels can influence the trajectory and severity of anxiety and depression. Patients with maladaptive coping mechanisms, such as avoidance or rumination, may experience persistent or exacerbated symptoms, whereas those with robust support networks and effective coping strategies are often better able to navigate the psychological challenges of their diagnosis [36,53].

## 4. Nutritional Status in Cervical Cancer

### 4.1. Malnutrition and Cancer Cachexia

Cancer cachexia, in particular, is a multifactorial syndrome driven by systemic inflammation, reduced food intake, and altered metabolism [54,55]. It is associated with decreased tolerance to treatment, increased risk of complications, and poorer survival outcomes, making it a key factor in disease progression.

Nutritional disturbances, including malnutrition and cancer cachexia, are frequently observed among patients diagnosed with cancer and represent a major clinical concern. These conditions are characterized by unintentional weight loss, progressive muscle wasting, and metabolic alterations, all of which contribute to a decline in functional status and overall health [56,57,58].

Evidence from the literature shows that a substantial proportion of oncology patients experience weight loss and nutritional deficiencies, often beginning at or even before diagnosis [59,60]. The prevalence and severity of these disturbances vary depending on tumor type and aggressiveness, with more advanced or aggressive malignancies being associated with higher rates of weight loss [56,61]. This suggests that nutritional impairment is not only a consequence of treatment but also an early manifestation of the disease process.

### 4.2. Nutritional Assessment Indicators

Assessing nutritional status is an important component of the clinical management of patients with cervical cancer, as nutritional status may influence treatment tolerance, recovery, and overall clinical outcomes [62,63]. Various tools and indicators are used to evaluate nutritional status, including anthropometric measurements (such as body weight and body mass index), biochemical markers, and validated screening instruments like the Mini Nutritional Assessment (MNA) or Prognostic Nutritional Index (PNI) [64,65,66].

Studies have demonstrated that a large proportion of cancer patients fall into categories of either malnutrition or risk of malnutrition when evaluated using these tools [67,68]. Poor nutritional status has been consistently linked to increased postoperative complications, reduced treatment tolerance, and lower survival rates [69]. For instance, patients with more favorable nutritional indices tend to have better clinical outcomes and fewer complications compared to those with impaired nutritional status [70,71].

### 4.3. Relationship Between Nutrition and Mental Health

An increasing body of research highlights a strong relationship between nutritional status and psychological well-being in cancer patients. Individuals with impaired nutritional status often report higher levels of anxiety, depression, and perceived pain compared to those with adequate nutrition [58,72]. However, evidence specifically examining the relationship between nutritional status and mental health in patients with cervical cancer remains limited, and findings from broader cancer populations should therefore be interpreted with caution when applied to this specific patient group.

Psychological factors such as distress, depression, and chronic pain may both contribute to and result from poor nutritional status, creating a complex, bidirectional relationship [72]. For example, persistent pain and emotional distress can reduce appetite and food intake, thereby worsening nutritional deficiencies. Conversely, malnutrition may exacerbate fatigue, mood disturbances, and overall quality of life [73,74,75].

Studies have also shown that patients with better nutritional status tend to experience lower levels of psychological distress and improved symptom control [76,77,78].

Although nutritional impairment and psychological distress have each been documented in cervical cancer populations, direct evidence examining their relationship specifically in women with cervical cancer remains limited. Therefore, findings derived from other cancer populations should be considered indirect or extrapolated evidence that supports the clinical plausibility of this relationship but does not establish a cervical cancer-specific association or causal pathway.

Given these associations, it is important to evaluate not only the nutritional condition but also the psychological status of patients at the time of diagnosis. Early, integrated interventions targeting both nutrition and mental health may help reduce symptom burden, improve quality of life, and potentially enhance treatment outcomes.

### 4.4. Dietary Interventions and Nutritional Supplementation in Cervical Cancer

As early as 1989, epidemiological studies suggested the existence of a relationship between diet and the risk of CC. A case–control study conducted in the Seattle area showed that a high intake of green and yellow vegetables, fruit juices, and nutrients such as carotenoids, vitamin C, and vitamin E was associated with a reduced risk of invasive cervical cancer. For example, women with a high intake of vitamin C had approximately a two-fold lower risk compared with those with low intake [79]. Building on these observations, more recent research has highlighted the mechanisms through which diet may influence the risk of cervical cancer. Nutrition plays an essential role in the functioning of the immune system and is a determining factor in the body’s ability to prevent and eliminate infections, including human papillomavirus (HPV) infection, the main etiological factor of cervical cancer [80]. Foods and specific nutrients may contribute to reducing the risk of progression from persistent high-risk human papillomavirus (hrHPV) infection to cervical cancer [81,82]. Notably, low folate intake has been associated with up to a nine-fold higher risk of CIN3+ lesions. Conversely, higher intake of certain vitamins and antioxidant-rich foods has consistently been correlated with lower persistence and progression of HPV infection [83], while folate supplementation has been shown to promote regression of CIN lesions [84].

Several case–control studies have investigated the association between diet and the risk of cervical cancer or CIN (Table 3). Barchitta et al. [81] showed that a Mediterranean-like dietary pattern, rich in legumes, vegetable soups, potatoes, raw and cooked vegetables, and olive oil, was associated with a lower risk of high-risk HPV infection and CIN2+, whereas a “Western” dietary pattern characterized by high consumption of chips, snacks, sauces, and processed and red meat increased the risk. In India, Rajkumar et al. [85] demonstrated that low consumption of vegetables and fruits was associated with a higher risk of invasive cervical cancer among women infected with HPV. De Vet et al. [86] found no evidence that β-carotene protects against cervical dysplasia, although vitamin C and dietary fiber showed a weak inverse association that was not statistically significant. Hosono et al. [87] reported that dairy products, fish, tofu, and green leafy vegetables were associated with a significant or marginally significant reduction in the risk of CIN3 and invasive cervical cancer among Japanese women. Tomita et al. [88] found a strong inverse association between the consumption of green and yellow vegetables, fruits, and serum levels of lycopene and tocopherol and the risk of CIN and invasive cancer.

In another study, Hwang et al. [89] confirmed the protective role of fruits and vegetables in reducing the risk of cervical dysplasia, particularly among HPV-positive women with a high viral load. Hernandez et al. [90] observed that some dietary sources of lignans, such as garlic and taro leaves, were associated with an increased risk of cervical intraepithelial lesions, while González et al. [91] suggested a protective role of fruits and other dietary factors in the European Prospective Investigation into Cancer and Nutrition study. Furthermore, Ghosh et al. [92] demonstrated that a diet rich in fruits, vegetables, and micronutrients such as fiber, vitamins C, E, and A, carotenoids, folate, and lycopene may reduce the risk of cervical cancer independently of other risk factors.

A case–control study conducted among women at risk of ovarian and cervical cancer assessed the intake of flavonoid-rich foods and found differences, particularly in berry consumption across BMI categories, highlighting the need for targeted nutritional interventions for prevention [93]. Nutritional status directly influences the immune response, and nutritional deficiencies may lead to a reduced ability to clear HPV infection, thereby promoting its persistence and progression to precancerous lesions and cancer. Certain micronutrients, such as vitamins A, C, and E, as well as folate, have been associated with the inhibition of cellular proliferation, protection of DNA, and support of immune function [80]. In addition to specific nutrients, the consumption of fruits and vegetables has been shown to play an important role in the prevention of cervical cancer [83]. Lower intake has been associated with a higher risk of hrHPV infection [94]. A study conducted in Iran reported that vitamin B1 (thiamine), together with vitamins B3 and B6, was associated with a lower risk of cervical cancer [95]. Overall, the evidence suggests that a diet rich in vegetables, fruits, dairy products, fish, and other antioxidant and anti-inflammatory nutrients may have a protective effect against HPV infection, precancerous lesions, and cervical cancer.

It is important to distinguish this evidence from studies addressing nutritional status and psychological outcomes in patients with established cervical cancer. Most of the studies summarized in this section investigated dietary exposure in relation to HPV persistence, cervical intraepithelial neoplasia, or cervical cancer risk, rather than anxiety or depression in women diagnosed with cervical cancer. Therefore, these findings provide cervical cancer-specific evidence regarding dietary and nutritional factors in disease prevention and progression, but they should not be interpreted as direct evidence of a relationship between nutrition and psychological distress in cervical cancer patients.

### 4.5. Minerals

Minerals, essential for regulating cellular proliferation and playing a critical role in a wide range of physiological processes necessary for maintaining health and preventing disease, are often overlooked. Dietary imbalances in minerals, which are common in industrialized countries, increase the risk of cancer [96,97,98]. Unlike vitamins, which are organic substances, minerals originate from the soil and cannot be produced by living organisms. They are classified into two categories depending on the amount required by the body: macrominerals, such as calcium (Ca), phosphorus (P), magnesium (Mg), sodium (Na), potassium (K), and chloride (Cl), which are needed in larger amounts, and trace elements, such as iron (Fe), zinc (Zn), copper (Cu), selenium (Se), chromium (Cr), iodine (I), manganese (Mn), and molybdenum (Mb), which are required in smaller quantities [97,99]. Most minerals act as cofactors in cell differentiation and have essential roles in supporting the immune system [83,100,101,102]. In the context of cancer, minerals contribute to the regulation of OS, immune system function, and the control of cellular proliferation, processes involved in tumor initiation and progression [96,100]. Deficiencies or excesses of certain minerals may influence cancer susceptibility and disease progression [103].

Calcium contributes to muscle contraction, nerve impulse transmission, cell communication, and the regulation of membrane permeability [97,104,105,106]. In addition, Ca plays an important role in controlling cell proliferation and differentiation and may influence apoptosis processes, which are essential mechanisms for preventing malignant transformation [107,108,109]. Through these mechanisms, adequate Ca intake may help reduce the risk of developing certain types of cancer. In a case–control study involving 405 cases of cervical neoplasia (333 invasive cancers and 72 CIN III) compared with 2025 controls, higher Ca intake was associated with up to a ~50% reduction in risk, while high vitamin D intake reduced the risk by ~64% [87] (Table 4). In a multi-institutional cross-sectional study including 1096 participants aged 18 to 65 years, Ca supplementation was significantly associated with a lower risk of CIN 2/3 (OR 0.21; 95% CI: 0.08–0.50), confirming the protective role of Ca in cervical health [94].

Magnesium is primarily regulated through intestinal absorption and renal reabsorption and acts as a cofactor in reactions involving adenosine triphosphate (ATP) [110]. It supports bone mineralization, maintains cellular electrolyte balance, and contributes to the regulation of immune responses by modulating nuclear factor kappa-light-chain-enhancer of activated B cells (NF-κB) and cytokine production [97,111,112]. Through its role in DNA stability, DNA repair, and inflammation regulation, Mg may influence processes involved in carcinogenesis [113,114]. Adequate Mg levels may contribute to reducing OS and maintaining genomic integrity [115,116].

Sodium is an essential electrolyte involved in the regulation of fluid balance, nerve function, and cellular homeostasis, making it a key factor in maintaining normal physiological processes [117,118,119]. Serum Na levels reflect fluid balance and influence extracellular fluid volume and blood pressure [97]. Although the direct role of Na in carcinogenesis is less clear, electrolyte imbalances may affect cellular homeostasis and physiological functions, and excessive Na intake has been indirectly associated with inflammatory processes and certain types of cancer [120].

Potassium is rapidly regulated through intra- and extracellular redistribution and, in the long term, through renal excretion. It supports cellular fluid balance, nerve excitability, muscle contraction, ATP production, and the maintenance of acid-base balance [97,121,122]. Additionally, K contributes to maintaining membrane potential and proper cellular function, and imbalances may influence cellular proliferation and programmed cell death mechanisms involved in preventing tumor development [123,124,125].

**Table 4:** Summary of studies investigating mineral intake and cervical cancer related outcomes.

Study	Sample Size	Minerals	Administrated	Duration	Results	References
Randomized Clinical Trial	80 (40 case/40 control), 21–55 y, HPV+ with abnormal Pap	Zn	220 mg every 12 h	3 months	Zn supplementation increased clearance of HR-HPV and regression of cervical cytology	[126]
Randomized Clinical Trial	104 (50 experimental/54 control)	Se	100 µg twice daily	5 weeks	Increased blood Se levels and reduced hematologic toxicity during chemoradiotherapy	[127]
Cohort Study	327, 18–60 y, HPV+	Fe	-	-	High ferritin associated with lower clearance of oncogenic and HPV-16 infections	[128]
Case–control	58 (29 poor prognosis/29 good prognosis)	Se	-	-	Low plasma Se associated with poor prognosis in cervical cancer	[129]
Case–control	90 (45 CIN/45 controls), ≤65 y	Se	-	-	Lower Se levels linked to more severe CIN III	[130]
Case–control	2430 (405 cervical neoplasias/2025 controls)	Ca/Vitamin D	-	-	Higher Ca intake ↓ risk ~50%; higher vitamin D intake ↓ risk ~36%	[87]
Multi-institutional Cross-sectional	1096, 18–65 y	Ca	-	-	Ca supplements associated with lower risk of CIN 2/3 (OR 0.21)	[94]

Ca—Calcium; CIN—Cervical Intraepithelial Neoplasia; Fe—Iron; HPV = Human Papillomavirus; HR-HPV—High-Risk Human Papillomavirus; OR—Odds Ratio; Se—Selenium; Zn—Zinc; ↓—decreased.

Iron is regulated through the hepcidin–ferroportin axis: hepatic hepcidin blocks Fe export by binding to ferroportin, thereby reducing its serum levels. Fe is an essential enzymatic cofactor and participates in oxygen transport, metabolism, biomolecule synthesis, ATP production, antioxidant functions, and defense against pathogens [97,131,132,133]. A cohort study involving 327 HPV-positive women aged 18 to 60 years showed that higher ferritin levels were associated with a lower likelihood of clearing oncogenic infections and HPV-16 compared with women with lower ferritin levels [128]. In oncology, Fe metabolism is particularly important because excess Fe may promote OS and the formation of free radicals, contributing to DNA damage and tumor progression, while deficiency may impair immune system function [134,135,136].

Zinc is primarily regulated through fecal excretion and is an essential enzymatic element involved in the metabolism of proteins, lipids, carbohydrates, and nucleic acids, as well as in gene expression regulation and cellular membrane stabilization [137,138]. Absorption occurs in the duodenum and jejunum and is positively influenced by chelators such as glutathione and amino acids and negatively influenced by an alkaline environment or competing minerals (Ca, Fe). Important sources include oysters, red meat, dairy products, legumes, and eggs. Deficiency causes gastrointestinal disorders, skin impairment, alopecia, vision problems, reduced protein synthesis, weakened immunity, and impaired glucose tolerance, and is assessed by measuring serum or plasma Zn levels [97,139,140]. Zn plays an essential role in DNA stability, the function of proteins involved in DNA repair, and immune system activity, thereby contributing to protection against malignant cellular transformation [141]. A randomized clinical study conducted on 80 women aged 21 to 55 years, all with a positive HPV test and abnormal cervical cytology, evaluated the effect of Zn supplementation. Participants were randomly assigned to case groups receiving 220 mg of Zn every 12 h for three months. The results showed that oral administration of Zn sulfate significantly increased the chances of clearing high-risk HPV infection and promoted the regression of cervical cytological abnormalities [126].

Selenium is primarily regulated through renal excretion and is essential for the synthesis of selenoproteins, antioxidant functions, maintenance of redox balance, repair of oxidative damage, and thyroid hormone metabolism [142,143]. Absorption occurs in the small intestine, and the recommended daily intake for adults is 55 µg. Major sources include red meat, fish, eggs, nuts, and dairy products [97]. Through its antioxidant role, Se protects cells against OS and DNA damage, processes involved in cancer initiation and progression. It may also influence immune response and the sensitivity of tumor cells to oncological treatments. Another randomized clinical study included 104 patients with cervical cancer divided into an experimental group (n = 50) and a control group (n = 54). All patients received standard treatment, including external pelvic irradiation, five cycles of chemotherapy, and brachytherapy. The experimental group received Se supplementation (100 µg twice daily) for five weeks. Se supplementation increased blood Se levels in deficient patients and significantly reduced the hematological toxicity associated with combined chemotherapy and radiotherapy treatment [127]. In a case–control study, 58 patients were analyzed according to prognosis (29 with poor prognosis and 29 with good prognosis). An association was identified between low plasma Se levels and a poorer prognosis of cervical cancer, suggesting the involvement of Se deficiency in GPL metabolism [129]. Another case–control study comparing 45 women with cervical intraepithelial lesions (CIN) with 45 women with normal cytology reported lower serum Se levels in patients with CIN, particularly in severe forms (CIN III: ~40.5 µg/L vs. 59.5 µg/L in the control group). The study suggests that lower Se levels are associated with more severe forms of CIN [130].

Chromium is primarily regulated through renal excretion and contributes to enhancing insulin action, although the exact mechanism is not fully elucidated [144]. Absorption occurs in the small intestine, and the recommended daily intake for adults ranges between 20–35 µg depending on age and sex. Main sources include fruits, vegetables, cereals, legumes, and meat. Deficiency may cause peripheral neuropathy, insulin resistance, and weight loss and is more frequent in hospitalized patients with malnutrition or parenteral nutrition without adequate supplementation, as well as in cancer patients after gastrointestinal surgery. There are no specific tests or consensus regarding optimal supplementation [97]. Through its influence on glucose metabolism and insulin sensitivity, Cr may have an indirect role in metabolic processes associated with the development of certain cancers [145].

Iodine is primarily regulated by the thyroid through the synthesis and storage of the hormones T4 and T3 in the follicular colloid, with controlled release by TSH and maintenance of homeostasis through hormonal effects and urinary excretion [146,147]. Rapidly absorbed in the stomach and partially in the duodenum, iodine is essential for the synthesis of thyroid hormones [146]. The recommended daily intake for adults is 150 µg, and main sources include iodized salt, fish, seaweed, eggs, dairy products, and chicken [97,148]. Thyroid hormones influence cellular metabolism, proliferation, and differentiation, processes also involved in cancer development [147].

Manganese is primarily regulated through biliary excretion and acts as an enzymatic activator and a component of metalloenzymes [149], participating in the synthesis of urea, bone, cartilage, and connective tissue, the metabolism of amino acids and carbohydrates, antioxidant activity, and the modulation of cellular signaling [97,150,151]. Absorption occurs in the small intestine, and the recommended daily intake is 2.3 mg for men and 1.8 mg for women [152]. Main sources include cereals, nuts, legumes, green vegetables, and fruits [153]. Mn is a component of the antioxidant enzyme mitochondrial superoxide dismutase (MnSOD), which protects cells against free radicals and contributes to preventing DNA damage associated with carcinogenic processes [154,155].

## 5. Oxidative Stress in Cervical Cancer

### 5.1. Pathophysiological Mechanisms

Oxidative stress plays a crucial role in the initiation and progression of cervical cancer, being defined as an imbalance between the production of reactive oxygen species (ROS) and the antioxidant defense systems of the body [14,156]. Under physiological conditions, ROS are involved in normal cellular signaling processes; however, excessive accumulation leads to damage of essential biomolecules, including DNA, lipids, and proteins [157,158].

In cervical cancer, persistent infection with high-risk human papillomavirus (HPV) is considered the primary etiological factor, and oxidative stress is closely linked to HPV-mediated carcinogenesis [159,160]. Viral oncoproteins such as E6 and E7 interfere with tumor suppressor pathways, including p53 and retinoblastoma (Rb) proteins, thereby promoting genomic instability. This disruption is further exacerbated by increased ROS production, which induces DNA mutations, chromosomal aberrations, and epigenetic alterations [161,162,163].

Chronic inflammation, often associated with persistent HPV infection, is another key contributor to oxidative stress. Inflammatory cells generate large amounts of ROS and reactive nitrogen species (RNS), creating a microenvironment that supports tumor development and progression [14,161,164]. Additionally, oxidative stress can activate multiple signaling pathways, such as NF-κB, MAPK, and PI3K/Akt, which are involved in cell proliferation, survival, angiogenesis, and resistance to apoptosis [165,166,167].

Mitochondrial dysfunction also plays a significant role in ROS overproduction in cancer cells. Altered mitochondrial metabolism leads to increased electron leakage and further ROS generation, reinforcing a cycle of oxidative damage. Moreover, cancer cells often exhibit impaired antioxidant defense mechanisms, including reduced activity of enzymes such as superoxide dismutase (SOD), catalase, and glutathione peroxidase [168,169,170].

### 5.2. Oxidative Stress Biomarkers

The assessment of oxidative stress in cervical cancer relies on the measurement of specific biomarkers that reflect the balance between oxidant production and antioxidant capacity. These biomarkers can be broadly categorized into indicators of oxidative damage and components of the antioxidant defense system [171,172].

Markers of lipid peroxidation, such as malondialdehyde (MDA) and 4-hydroxynonenal (4-HNE), are frequently used to evaluate oxidative damage to cell membranes [173]. Elevated levels of these compounds have been associated with increased oxidative stress and have been reported in patients with various types of cancer, including cervical cancer [174,175,176].

Protein oxidation can be assessed through the measurement of protein carbonyls and advanced oxidation protein products (AOPP), which indicate structural and functional alterations of proteins caused by ROS [177]. Similarly, oxidative damage to DNA is commonly evaluated using biomarkers such as 8-hydroxy-2′-deoxyguanosine (8-OHdG), which reflects oxidative modifications in DNA and is considered a reliable indicator of mutagenic processes [178].

In addition to damage markers, antioxidant status is evaluated by measuring both enzymatic and non-enzymatic components. Key antioxidant enzymes include superoxide dismutase (SOD), catalase (CAT), and glutathione peroxidase (GPx), all of which play essential roles in neutralizing ROS [179,180]. Non-enzymatic antioxidants, such as reduced glutathione (GSH), vitamins C and E, and other dietary antioxidants, also contribute significantly to maintaining redox balance [181,182].

Total antioxidant capacity (TAC) is another important parameter, providing an overall estimate of the body’s ability to counteract oxidative stress. In many cancer patients, decreased antioxidant levels combined with elevated oxidative damage markers indicate a disrupted redox homeostasis [183,184].

### 5.3. Potential Relationship Between Oxidative Stress and Psychological Distress

Beyond its established role in cervical carcinogenesis, oxidative stress has also been investigated in relation to psychological disorders, particularly depression. A meta-analysis of 23 observational studies including 4980 participants found that depression was associated with increased oxidative stress and reduced antioxidant status, although substantial heterogeneity was observed across studies [185]. Similarly, Black et al. reported in a systematic review and meta-analysis that depression was associated with increased levels of the oxidative damage markers 8-hydroxy-2′-deoxyguanosine (8-OHdG) and F2-isoprostanes [186]. Further meta-analytic evidence showed that depressive disorders were associated with alterations in several oxidative stress parameters, including increased malondialdehyde (MDA) and reduced total antioxidant capacity (TAC) [187]. These findings are of potential relevance to the present review because MDA, 8-OHdG, and antioxidant defense parameters are also commonly investigated as indicators of redox imbalance in cervical cancer.

However, an important distinction must be made between this broader evidence and cervical cancer-specific evidence. The studies linking oxidative stress biomarkers with depression were predominantly conducted in psychiatric or general clinical populations rather than specifically in women with cervical cancer [185,186,187]. Direct studies simultaneously assessing oxidative stress biomarkers and validated measures of anxiety or depression in cervical cancer patients remain scarce. Therefore, the overlap in oxidative stress markers provides biological plausibility for a potential relationship between redox imbalance and psychological distress but does not establish a cervical cancer-specific causal or mediating pathway. Prospective studies simultaneously evaluating oxidative stress biomarkers and standardized psychological outcomes are needed to determine whether this association is present and clinically relevant in patients with cervical cancer.

## 6. Clinical Implications

The findings summarized in this review highlight several important considerations for the clinical management of patients with cervical cancer. Anxiety and depression are highly prevalent and can emerge at any stage, from diagnosis through treatment and into survivorship. Recognizing and addressing these mental health issues is crucial, as untreated psychological distress can compromise treatment adherence, intensify physical symptoms such as pain and fatigue, and negatively affect overall quality of life. Clinicians should therefore incorporate routine psychological assessment into oncology practice, employing validated instruments such as the Hospital Anxiety and Depression Scale (HADS) or other standardized tools to identify patients at risk.

Equally important is the evaluation of nutritional status. Malnutrition and cancer-related cachexia are common among cervical cancer patients and are associated with poorer treatment tolerance, higher rates of complications, and diminished survival. Early nutritional screening and intervention can improve functional status, support immune function, and enhance both physical and psychological resilience. Interventions may include individualized dietary counseling, oral nutritional supplementation, or, where indicated, referral to specialized dietetic services.

The interplay between oxidative stress and psychological health further emphasizes the need for a holistic approach. Excessive reactive oxygen species (ROS) production and compromised antioxidant defenses not only promote disease progression but may also contribute to neuropsychological symptoms, including fatigue, cognitive impairment, and mood disturbances. Assessing oxidative stress markers and considering antioxidant strategies—either dietary or pharmacological—could complement standard oncological care, though further research is required to establish clinically effective protocols.

Finally, multidisciplinary collaboration is essential. Optimal care involves coordination between oncologists, psychologists, dietitians, and nursing staff to provide integrated support that addresses both the somatic and emotional needs of patients. Psychoeducational programs, social support networks, and interventions that enhance coping skills can mitigate anxiety and depression, while nutritional and oxidative stress management can improve physical resilience. By adopting such a comprehensive strategy, clinicians may enhance not only survival outcomes but also the overall quality of life for cervical cancer patients.

## 7. Conclusions

Cervical cancer is associated with a substantial psychological burden, particularly anxiety and depression, which can negatively affect quality of life, treatment adherence, and overall clinical outcomes. Nutritional impairment, including malnutrition and cancer-related cachexia, may further contribute to fatigue, functional decline, and psychological vulnerability, while emotional distress may adversely affect appetite and nutritional intake. These findings support the importance of integrating psychological and nutritional assessment into comprehensive cervical cancer care.

Oxidative stress is well established in the pathophysiology and progression of cervical cancer; however, direct evidence linking oxidative stress to psychological outcomes in this specific population remains limited. Therefore, although redox imbalance may represent a potential biological pathway connecting disease-related processes, nutritional impairment, and psychological vulnerability, this relationship should currently be considered hypothesis-generating rather than an established causal mechanism. Overall, the available evidence suggests potential interactions among psychological, nutritional, and oxidative factors, but studies directly evaluating all three domains in cervical cancer are still lacking.

Future longitudinal and interventional studies are needed to clarify these relationships and determine whether integrated approaches targeting psychological distress, nutritional status, and oxidative imbalance can improve patient outcomes. A multidisciplinary approach incorporating psychological support, nutritional assessment, and individualized supportive care may contribute to improved quality of life and overall well-being in patients with cervical cancer.

## Figures and Tables

**Table 1:** Prevalence of Anxiety in Cervical Cancer Patients.

Study Design	Sample Size	Assessment Tool	Key Findings	References
Observational (case–control)	158 patients + 150 controls	HADS (Hospital Anxiety and Depression Scale)	Anxiety levels were significantly higher in cervical cancer patients compared to controls; associated with poorer survival outcomes.	[32]
Cohort study	4245 patients	Psychological distress measures	Psychological distress (including anxiety) was associated with increased cancer-specific mortality.	[33]
Cross-sectional	269 patients	SOC, PSS, anxiety/depression scales	High prevalence of anxiety; psychosocial factors (social support, coping) significantly influenced anxiety levels.	[34]
Cross-sectional	103 patients	QoL & psychological scales	High prevalence of anxiety (~60%); associated with fatigue, insomnia, and poor quality of life.	[35]
Cross-sectional	59 patients	Zung Self-Rating Anxiety Scale (SAS)	Anxiety severity increased with advancing cancer stage.	[36]
Cross-sectional	200 patients	Psychological scales	Anxiety prevalence ~39.5%; associated with illness uncertainty.	[37]
Observational	150 patients	Anxiety & resilience scales	Higher resilience and social support were linked to lower anxiety levels.	[38]

HADS—Hospital Anxiety and Depression Scale, SOC—Sense of Coherence; PSS—Perceived Stress Scale; QoL—Quality of Life; SAS—Zung Self-Rating Anxiety Scale.

**Table 2:** Prevalence of Depression in Cervical Cancer Patients.

Study Design	Sample Size	Assessment Tool	Key Findings	References
Observational (case–control)	158 patients + 150 controls	HADS-D (Hospital Anxiety and Depression Scale)	Higher prevalence and severity of depression in cervical cancer patients compared with controls; associated with worse overall survival.	[32]
Cross-sectional	59 patients	Zung Depression Scale (SDS)	Depression scores were increased in all cervical cancer patients and correlated with disease stage.	[37]
Cross-sectional	224 patients	HADS and psychosocial scales	Prevalence of depression ~52.2%; high co-occurrence with anxiety; psychosocial resources (hope, optimism) predicted lower depression.	[6]
Meta-analysis	5681 patients (26 studies)	Various validated instruments	Pooled prevalence of depressive symptoms ~46% during peri-chemoradiotherapy period.	[40]
Cross-sectional	264 patients	HAMD/Zung/perceived support scales	Perceived social support and sense of coherence were negatively associated with depressive symptoms.	[45]

HADS-D—Hospital Anxiety and Depression Scale, Depression subscale; SDS—Zung Self-Rating Depression Scale; HAMD—Hamilton Depression Rating Scale.

**Table 3:** Case–control studies examining dietary factors in relation to cervical cancer and cervical lesions.

Type of Study	Outcome	Characteristics	Participants	Measure	Main Finding	Ref.
Case–control	cervical cancer	Mediterranean vs. Western dietary patterns	539 women (160 CIN1, 57 CIN2, 67 CIN3, 3 CIS)	Mediterranean diet score	Mediterranean diet reduced high-risk HPV infection and CIN2+	[81]
		Western/unhealthy dietary pattern: high chips, snacks, dipping sauces, vegetable oils, processed/red meat, low olive oil		Dietary pattern analysis	Associated with ↑ risk of high-risk HPV infection and ↑ risk of CIN2+	
Case–control	cervical cancer	Vegetables and fruits: green vegetables, tomatoes, carrots, cruciferous	205 ICC, 213 controls	Face-to-face FFQ	Low intake ↑ risk of ICC in HPV-infected women	[85]
Case–control	Cervical dysplasia	Spinach, fruits, tomatoes	Spinach, fruits, tomatoes	Postal questionnaire	β-carotene not protective; vitamin C and fiber had weak inverse association	[86]
Case–control	Cervical neoplasias	Dairy, fish, tofu, green leafy vegetables	333 invasive carcinoma, 72 CIN3, 2025 controls.	semiquantitative food-frequency questionnaire (FFQ).	Calcium and vitamin D ↓ neoplasia risk; dairy ↓ invasive carcinoma; fish, tofu, leafy greens ↓ CIN3 (marginal)	[87]
Case–control	CIN 1–3	Dark green and deep yellow vegetables, fruits, carrots	453 controls, 523 cases (CIN1, n 5140; CIN2, n 5126; CIN3, n 5231; invasive cancer, n 5108)	Validated FFQ and biochemical measurements	Strong inverse association with CIN and invasive cancer	[88]
Case–control	CIN 1–3	Vegetables and fruits	328 HPV-positive women (166 controls, 90 CIN I and 72 CIN II/III)	Lifestyle and dietary questionnaire	Fruit and vegetable intake ↓ risk of dysplasia and cancer, especially in HPV-positive women with high viral load	[89]
Case–control	SIL	Milk, yogurt, fish, tofu, green leafy vegetables	122 cases, 183 controls	Questionnaire and blood measurements of isoflavonoids, flavonoids, lignans	Dietary lignans (garlic, taro leaves) ↑ SIL risk	[90]
Case–control	cervical cancer and invasive squamous cervical cancer	Vegetables and fruits	299,649 women (EPIC)	Dietary questionnaire	Possible protective effect of fruit and other dietary factors	[91]
Case–control	Squamous cell carcinoma	Selected nutrients, fruits, vegetables	239 cases, 979 controls	Self-administered questionnaire	Diet high in fruits, vegetables, fiber, and vitamins ↓ cervical cancer risk	[92]

CIN—Cervical Intraepithelial Neoplasia; EPIC—European Prospective Investigation into Cancer and Nutrition; FFQ—Food-Frequency Questionnaire; HPV—Human Papillomavirus; ICC—Invasive Cervical Cancer; SIL—Squamous Intraepithelial Lesion; ↑—increased; ↓—decreased.

## Data Availability

No new data were created or analyzed in this study. Data sharing is not applicable to this article.
